# Bolus-Tracked Biphasic Contrast-Enhanced CT Imaging Following Microwave Liver Ablation Improves Ablation Zone Conspicuity and Semi-automatic Segmentation Quality

**DOI:** 10.1007/s00270-024-03948-x

**Published:** 2025-01-09

**Authors:** Louise Giansante, Ed McDonagh, Jodie Basso, Arafat Haris, Sajjan KC, Samuel J. Withey, Joshua Shur, Nicos Fotiadis, S. Nahum Goldberg, Edward W. Johnston

**Affiliations:** 1 Joint Dept. of Physics, The Royal Marsden, 203 Fulham Road, London, SW36JJ UK; 2 Interventional Radiology, The Royal Marsden, 203 Fulham Road, London, SW36JJ UK; 3 Diagnostic Radiology, The Royal Marsden, 203 Fulham Road, London, SW36JJ UK; 4 Department of Radiotherapy and Imaging, Institute of Cancer Research, London, UK; 5 Hadassah Hebrew University Medical Center, Ein Karem, Jerusalem, Israel; 6 Beth Israel Deaconess Medical Center, Harvard Medical School, Boston, MA 02215 USA

**Keywords:** Tomography, X-Ray Computed, Liver, Ablation techniques, Image enhancement

## Abstract

**Purpose:**

Contrast-enhanced CT (CECT) may be performed immediately following microwave liver ablation for assessment of ablative margins. However, practices and protocols vary among institutions. Here, we compare a standardized bolus-tracked biphasic CECT protocol and compare this with a single venous phase fixed delay protocol for ablation zone (AZ) assessment.

**Methods:**

An institutional review board approved study performed at a specialist cancer centre. A prospective cohort of patients undergoing bolus-tracked biphasic imaging was compared with a retrospective cohort of patients who underwent fixed delay venous phase imaging. AZ conspicuity and segmentation quality were semi-quantitatively scored using Five-point Likert scales. Time between ablation and image acquisition was recorded for each AZ and was correlated to AZ conspicuity and segmentation quality.

**Results:**

Forty patients, median age 59 years (IQR 48–66 years), 24 men, underwent microwave ablation of 68 liver tumours. AZ conspicuity was higher in the bolus-tracked (*n* = 33) vs. fixed delay (*n* = 35) cohorts, 4.5 vs. 2.5, *P* < 0.0001. Commensurate segmentation quality was also higher, 5.0 vs. 3.0 respectively, *P* < 0.0001. Ordinal regression showed that image quality scores declined by 3–4% for each minute that passes after ablation, particularly for arterial phase images, where regression coefficients were − 0.04, *P* = 0.007, and -0.03, *P* = 0.012 for conspicuity and segmentation quality, respectively.

**Conclusion:**

Bolus-tracked biphasic contrast-enhanced CT protocols improve both conspicuity and semi-automatic segmentation quality of microwave liver ablation zones, particularly if imaged soon after ablation.

**Evidence-Based Medicine:**

Level 2b; exploratory prospective cohort study.

**Graphical Abstract:**

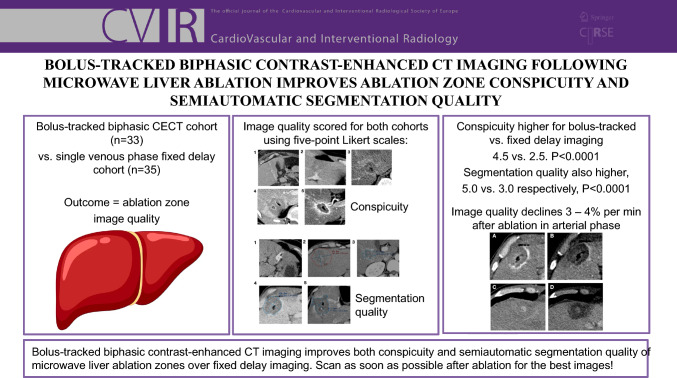

## Introduction

Thermal ablation is a well-established minimally invasive treatment for both primary and oligometastatic liver tumours. When judiciously employed, ablation can eradicate tumours [1, 2] by completely surrounding tumours by an ablation zone (AZ) including an ample ‘safety margin’ of surrounding liver parenchyma, typically > 5 mm [3, 4].

Reliable margin adequacy judgement depends on adequate imaging, with recent guidelines recommending contrast-enhanced CT [5]. To judge the relationship between tumours and AZs, pre- and post-ablation images may either be assessed visually in a ‘side-by-side’ comparison [6], or – ideally – using ablation confirmation software[7]. This latter approach requires accurate tumour and AZ segmentation, registration and fusion, for unbiased, objective, three-dimensional, quantitative assessment.

Nevertheless, significant practice variation persists among operators and centres. For instance, a survey of liver ablation in the United Kingdom revealed 49% always, 16% sometimes, and 35% never give contrast on post-ablation CT scans [8]. Even if contrast is administered, there is no consensus regarding optimal acquisition protocols. Furthermore, we have observed that AZs are more conspicuous when imaged sooner after ablation. Lack of standardization causes variations that hinder optimal outcomes [9–11]. While biphasic imaging protocols for *tumour* conspicuity are well-established [12], AZs are physiologically distinct and may not be adequately captured by such protocols. Therefore, specific focus upon AZ imaging is necessary.

In this study, we compare a prospective cohort of patients undergoing bolus-tracked biphasic contrast-enhanced CT (CECT) imaging with a retrospective cohort who underwent single venous phase fixed delay imaging for microwave liver AZ assessment. Our hypothesis was that bolus-tracked biphasic imaging improves ablation zone conspicuity and semi-automatic segmentation quality. We also seek to characterise any relationship between the timing of imaging and AZ depiction.

## Materials and Methods

### Study Cohorts

This study was approved by our institutional review board (7/2/2022, identifier 1155), who waived the need for individual informed written consent, as CECT is part of standard of care. A prospective ‘bolus-tracked cohort’ of patients underwent bolus-tracked *biphasic* CECT imaging between January and May 2023. This was compared with a retrospective ‘fixed delay cohort’ who underwent fixed delay *venous phase only* CECT imaging between October 2021 and February 2022. Procedures were performed at a single institution, The Royal Marsden Hospital, a specialist cancer centre in London. We received no funding for the study.

### Eligibility

Inclusion criteria were: i) patients undergoing microwave ablation of liver metastases; and ii) post-ablation CECT imaging under general anaesthesia. Exclusion criteria were: i) severely degraded imaging due to artefact or noise; and ii) inability to administer intravenous contrast (e.g., due to renal dysfunction or allergy).

### Ablation Procedures

Microwave ablation was performed by our team of four board certified interventional radiologists (6–24 years’ experience with liver ablation). Procedures were carried out under general anaesthesia in a 128-slice CT scanner (Siemens Definition Edge, Erlangen, DE) using high frequency jet ventilation (Monsoon Acutronic Jet ventilation system III, Hirzel, CH) to minimize respiratory excursion. Two microwave generator systems were used (NeuWave, Johnson & Johnson, WI, or Solero, Angiodynamics, NY).

Each patient received contrast medium twice, once for ablation planning and then immediately after ablation.

### Bolus-Tracked Biphasic CECT Protocol

For each scan, Omnipaque 350 mgI/mL (GE Healthcare, Bucks, UK), median 95mls (IQR 80–96mls) was injected through a ≥ 18G peripheral intravenous cannula at ≥ 5mls/s. A 50 ml saline flush was then administered at 5 mL/s. Monitoring began 15 s after injection. A helical scan of the whole liver was acquired. Table 1 presents full scan parameters.

**Table 1:** Bolus-tracked protocol scan parameters

Parameter	Late arterial phase	Portal venous phase
Mode	Spiral	Spiral
kV	120	120
Q_ref_ mAs*	170	170
Rotation time	0.5 s	0.5 s
Pitch	0.6	0.6
Detector configuration	128 × 0.6 mm	128 × 0.6 mm
Delay	12 s from aorta ≥ 200 HU	> 45 s from aorta ≥ 200 HU ^**^
Reconstruction kernel	B30f	B30f
Slice thickness	3 mm	3 mm
Slice interval	3 mm 1 mm for ablation planning	3 mm
Contrast volume	80 – 96 ml Omni 350 ≥ 5 ml/s	n/a

kV: kilovolts, mAs; milliamp seconds, Omni: Omnipaque

^*^Quality reference mAs (Q_ref_ mAs) is the effective mAs (mA * rotation time / pitch) required to achieve a specific image quality for a reference sized phantom. Tube current modulation subsequently adjusts the mAs according to individual patient sizes, using this image quality parameter as a reference point

^**^Considers the 12 s delay for the late arterial phase and approximately 5 s for the CT scanner table to transition between late arterial and portal venous acquisitions for a medium-sized patient

Twenty consecutive patients underwent the bolus-tracked protocol. Time from ablation completion to imaging was recorded for each AZ.

### Fixed Delay Cohort

Twenty consecutive patients underwent single venous phase ‘fixed delay’ post-ablation helical CECT scanning, with variable timing delays ranging from 65 to 90 s, based upon rough estimation of cardiac output. Omnipaque 300 was injected at 3 ml/sec. Acquisition parameters matched the bolus-tracked cohort.

### Assessment of Ablation Zone Conspicuity and Segmentation Quality

Two attending radiologists (JS and SW with 11 and 9 years’ experience respectively) who were blinded to imaging protocols and unaware of study design independently assessed AZ conspicuity on fully anonymized images (including study dates), with applicator locations on intraprocedural images available for AZ localisation. Readers assessed and scored the conspicuity of each AZ in the bolus-tracked cohort (arterial/venous phases) and fixed delay cohorts (*n* = 33/35), displayed randomly using the following standardised five-point Likert scale:

1. Very poor quality: cannot see the AZ border.
2. Poor quality/conspicuity of AZ. Somewhat helpful.
3. Satisfactory quality/conspicuity of AZ. Helpful.
4. Good quality/conspicuity of AZ. Very clear.
5. Excellent quality/conspicuity of AZ. Exemplary.

**Fig. 1:**
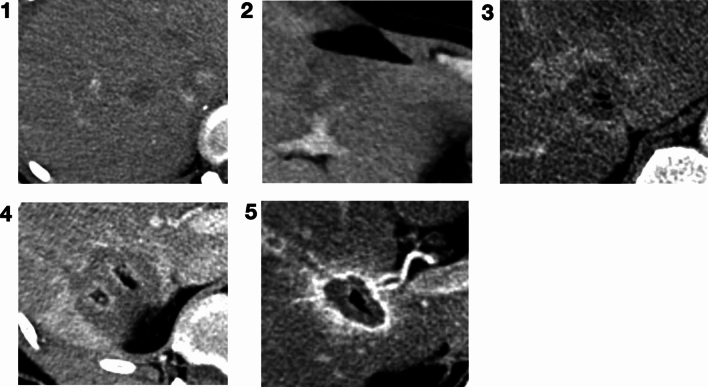
Examples of AZ conspicuity Likert scores (1 – 5) in the arterial phase

**Fig. 2:**
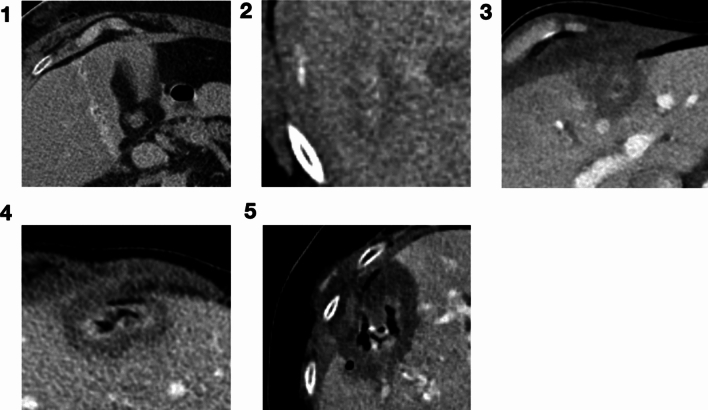
Examples of AZ conspicuity Likert scores (1 – 5) in the venous phase

Typical examples of AZ conspicuity Likert scores are shown in Figs. 1 and 2, for arterial and venous phases, respectively.

Semi-automatic AZ segmentation quality, without manual adjustment, was performed and assessed by JS using the ‘interpolated VOI’ tool on Mint Lesion™ (Mint Medical GmbH, Heidelberg, DE). This tool utilises a semi-automatic segmentation algorithm to delineate anatomical structures in three dimensions. Users define the structure's rough extent, aided by a boundary sphere display, and the algorithm identifies object boundaries across image slices [13–15]. The quality of AZ segmentation was scored using a five-point Likert scale, reflecting both performance of the automated algorithm and the extent of manual intervention required.

1. Non-diagnostic: AZ border is invisible.
2. Very poor quality: cannot segment the AZ border, only possible with manual segmentation.
3. Poor quality: segments < 50% of the AZ border.
4. Good quality: segments 50–90% of the AZ border.
5. Excellent quality: segments > 90% of the ablation border.

**Fig. 3:**
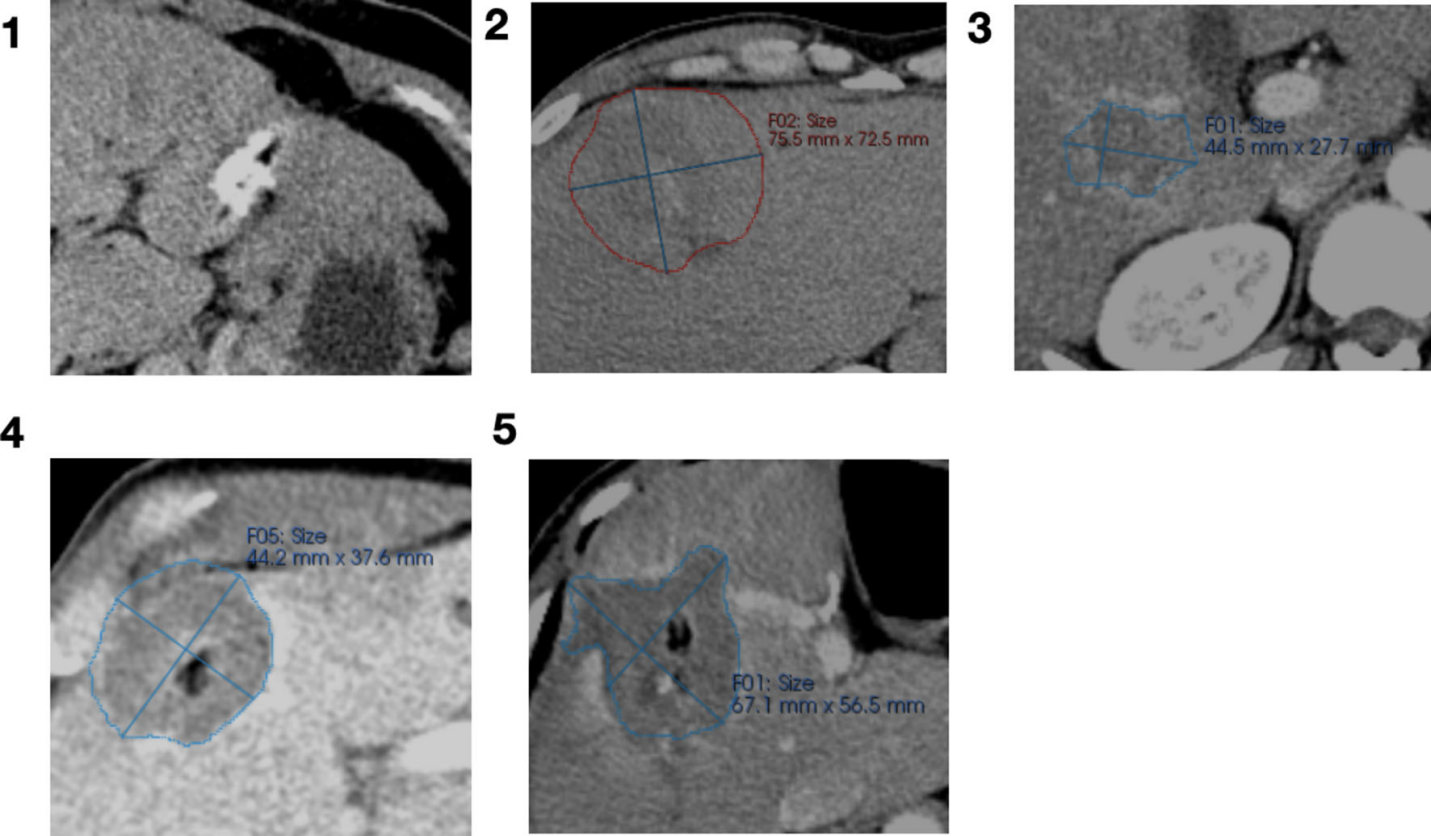
Examples of segmentation quality Likert scoring (1 – 5)

Typical examples are shown in Fig. 3.

### Study Design and Power Calculation

Although we initially intended to conduct a prospective randomised study, due to a marked improvement in image quality after the first few exploratory triggered biphasic scans, we considered it unethical to continue to prospectively subject patients to fixed delay scans. We therefore opted for a comparative analysis of prospective and retrospectively gathered data. Given the absence of established semi-quantitative scoring systems for liver ablation imaging, we were unable to perform an a priori sample size calculation. After analysis at 20 patients per cohort as a pragmatic decision, the data showed a large and highly significant effect size.

Initial *post-hoc* power analyses comparing the image quality of all 35 ablation zones of the fixed delay cohort versus 33 ablation zones in the biphasic cohort showed Cohen’s *d* = 1.76 and 1.14 for visual assessment and segmentation, respectively, conferring 99% power (1− *β*) in both groups at *α* = 0.05. To account for clustering effects from to multiple AZs in individual patients, we calculated Intra-Class Correlation Coefficients (ICCs) using linear mixed-effects models. Adjustments gave effective sample sizes of 24.8 and 25.5 (visual) and 32.6 and 28.5 (segmentation) for fixed delay and bolus-tracked cohorts, respectively. Final power calculations confirmed > 99% power at *α* = 0.05 for both assessments.

Baseline patient characteristics comprised age, sex, cancer type and prior treatments.

Outcome measures comprised:

(i) Comparison of conspicuity and segmentation quality scores for each AZ in bolus-tracked (whichever was higher of the arterial or venous phases) versus fixed delay cohorts.
(ii) Comparison of conspicuity and segmentation quality scores for each AZ in the venous phase of the bolus-tracked cohort, versus the fixed delay cohort; to enable like-for-like comparison of venous phase image quality.
(iii) Comparison of conspicuity and segmentation quality scores for arterial and venous phase imaging of the bolus-tracked cohort, to assess whether phases provide complimentary information.
(iv) Investigation of any relationship between acquisition time since ablation versus conspicuity and segmentation quality scores.
(v) Interobserver agreement of AZ conspicuity scoring.

### Statistical Analysis

Data were analysed using GraphPad Prism Version 9.4.1 (San Diego, CA), RStudio (Version 2024.04.02, PBC, Boston, MA) and Python 3.8 (https://www.python.org). Statistical significance was set at *P* < 0.05 and data were checked for normality using the Shapiro–Wilk test. Differences in unpaired variables were compared using independent sample t-tests or Mann–Whitney U tests, and differences in paired variables were compared using Wilcoxon testing. Concordance was assessed using Lin’s Concordance Correlation coefficient, ρ_c_ [16]_,_ interpreted according to McBride [17].

The relationship between the time between ablation and image acquisition upon conspicuity and segmentation quality scores was assessed using ordinal regression. Interobserver reliability was analysed using weighted kappa [18] and the mean of both readers was used for conspicuity scores.

## Results

### Baseline Patient Characteristics

Overall, forty patients, median age 59 years (interquartile range, IQR 48 – 66 years), 24 men, underwent microwave ablation of 68 target liver tumours, mainly colorectal cancer metastases (31/40, 78%).

Full baseline characteristics are shown in Table 2.

**Table 2:** Baseline characteristics for bolus-tracked and fixed delay cohorts

	Overall (n = 40)	Bolus-tracked cohort (n = 20)	Fixed delay cohort (n = 20)	P-value
Median age, yrs (IQR)	59 (48—66)	56 (44–67)	60 (53—65)	0.49
Men (%)	24/40 (60%)	11/20 (55%)	13/20 (65%)	0.52
Women (%)	16/40 (40%)	9/20 (45%)	7/20 (35%)	
CRC (%)	31/40 (78%)	16/20 (80%)	15/20 (75%)	0.70
Non-CRC (%)	9/40 (23%)	4 total:	5 total:	
			2 LMS	
			1 melanoma lung, breast	
		1 pancreas, H&N, LMS, NET		
Prior chemotherapy (%)	39/40 (97%)	19/20 (95%)	20/20 (100%)	0.31
Prior liver radiotherapy (%)	1/40 (2%)	0/20 (0%)	1/20 (5%)	0.31
Prior liver ablation (%)	20/40 (48%)	9/20 (45%)	11/20 (55%)	0.53
Prior liver surgery (%)	18/40 (43%)	8/20 (40%)	10/20 (50%)	0.52
Total number of AZs	68	33	35	0.92
	1 = 24	1 = 12	1 = 12	
	2 = 9	2 = 5	2 = 4	
	3 = 5	3 = 2	3 = 3	
	4 = 1	4 = 0	4 = 1	
	5 = 1	5 = 1	5 = 0	

*AZ* ablation zone, *GIST* gastrointestinal stromal tumour, *H&N* head and neck cancer, *NET* Neuroendocrine tumour, *LMS* leiomyosarcoma

### Bolus-Tracked vs. Fixed Delay Scans: Overall

The highest AZ conspicuity Likert score, whichever was higher of the arterial and venous phases, was 4.5 for the bolus-tracked vs. 2.5 for fixed delay cohort, *P* < 0.0001 (*n* = 68). Commensurate segmentation quality scores were 5.0 vs. 3.0, *P* < 0.0001 respectively.

### Bolus-Tracked vs. Fixed Delay Scans: Venous Phase

The highest AZ conspicuity Likert score was 4.0 vs. 2.5 for bolus-tracked venous phase and fixed delay scans respectively, *P* < 0.0001 (*n* = 68). Commensurate segmentation quality scores were 4.0 vs. 3.0, *P* = 0.002, respectively.

### Concordance of Arterial and Venous Phase Bolus-Tracked Imaging Components

Concordance, ρ_c_, of Likert scores of AZs in arterial vs. venous phases of the bolus-tracked cohort (n = 33) was 0.49 (95% CI 0.21–0.70) for conspicuity and 0.51 (0.22 – 0.71) for segmentation quality i.e., poor. A breakdown of how frequently arterial vs. venous phase imaging was preferred is shown in Table 3.

**Table 3:** AZ conspicuity and segmentation quality Likert scores

	Overall	< 30 min	> 30 min
*Visual conspicuity*			
Arterial preferred	14/33 (42%)	11/22 (50%)	3/11 (27%)
Venous preferred	13/33 (39%)	7/22 (32%)	6/11 (55%)
Neither preferred	6/33 (18%)	4/22 (18%)	2/11 (18%)
*Segmentation quality*			
Arterial preferred	12/33 (36%)	9/22 (41%)	3/11 (27%)
Venous preferred	6/33 (18%)	2/22 (9%)	4/11 (36%)
Neither preferred	15/33 (45%)	11/22 (50%)	4/11 (36%)

### Impact of Acquisition Time Post-Ablation upon AZ Depiction

**Fig. 4:**
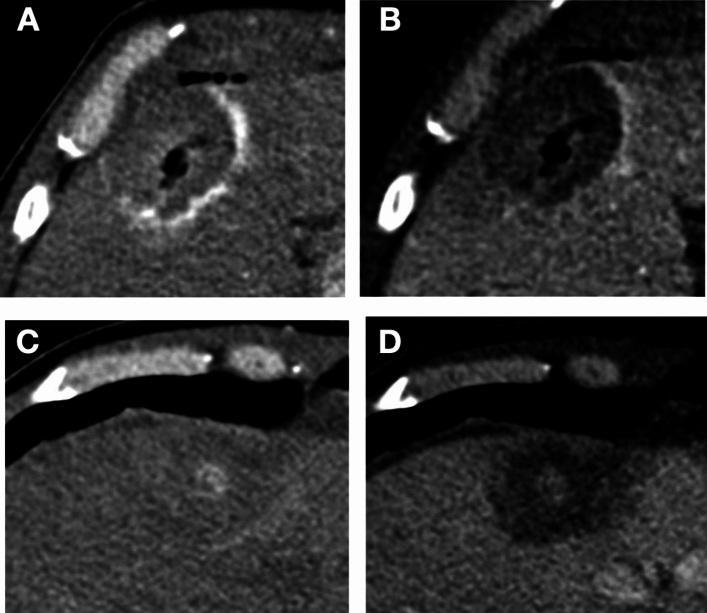
Diminishing ablation zone conspicuity as time passes after ablation demonstrated in the same 43-year-old woman who underwent microwave ablation for five medullary thyroid cancer liver metastases. **A:** Arterial phase image of the most recent AZ approximately 8 min after ablation, showing high conspicuity of the enhancing AZ periphery. **B:** Venous phase image of the same AZ as in A, showing high conspicuity of the nonenhancing central AZ. **C:** Arterial phase images of the least recent AZ in the same scan, approximately 90 min after ablation showed poor conspicuity of both the peripheral and central AZ. **D:** Venous phase images of the same AZ showed higher conspicuity of the central AZ. Findings suggest imaging is more useful when performed sooner after ablation, which is particularly true for arterial phase imaging

Thirty-three AZs were imaged with a median time of 13 min after ablation (IQR 8–41 min). When considering the time from the last ablation in each procedure, the median was 8 min (IQR 6–11 min). Imaging at extended timeframes from the ablation (> 1 h) reduced conspicuous peripheral AZ enhancement on arterial phase imaging. A typical example of this time-dependent enhancement behaviour is shown in Fig. 4.

Likert scores for arterial versus venous phase AZ imaging are shown in Table 3, including a breakdown for ablation zones imaged sooner (< 30 min, n = 22) and later (> 30 min, *n* = 11) after ablation.

Ordinal regression showed AZ conspicuity and segmentation quality scores decline as time elapses between ablation and imaging (Table 4).

**Table 4:** Ordinal regression results of image quality scores versus time between ablation and imaging in the bolus-tracked cohort

Predictor: Time since ablation (minutes)	Coefficient	Std. Error	*t*-value	*P*-value	*R*^2^
Visual conspicuity: arterial phase	− 0.04	0.01	− 2.70	0.007	0.31
Visual conspicuity: venous phase	− 0.03	0.01	− 2.10	0.036	0.20
Segmentation: arterial phase	− 0.03	0.01	− 2.50	0.012	0.30
Segmentation: venous phase	− 0.01	0.01	− 0.60	0.541	0.05

### AZ Conspicuity Scoring: Interobserver Reliability

Weighted kappa, κ, for interobserver conspicuity agreement was 0.80 (substantial agreement) for bolus-tracked arterial phase imaging, 0.50 for bolus-tracked venous phase imaging (moderate agreement) and 0.62 (substantial agreement) for the fixed delay (venous phase only) cohort.

## Discussion

This study demonstrates that a bolus-tracked biphasic contrast-enhanced CT (CECT) protocol significantly and substantially improves the conspicuity and segmentation quality of ablation zones (AZs) following microwave liver ablation. First and foremost, we found that visually assessed and semi-quantitatively scored AZ conspicuity was significantly higher for bolus-tracked biphasic than fixed delay imaging (4.5/5 vs. 2.5/5, *P* < 0.0001), as were segmentation quality scores (5.0 vs. 3.0 respectively, *P* < 0.0001). Agreement was moderate or substantial (κ = 0.50–0.80) amongst independent readers. Our data therefore suggest bolus-tracked biphasic imaging can dramatically and consistently improve AZ depiction. Paolucci et al. reported that bolus-tracked biphasic CECT protocols performed *before* ablation was associated with better outcomes [19]. Together, our studies therefore support using bolus-tracked protocols both before and after ablation.

Peripheral hypervascularity has also been observed on CT after RF ablation[20]. Although likely due to hyperacute thermally induced arterial vasodilatation, direct study is limited. Moreover, we found poor concordance (ρ_c_ = 0.49–0.51) of AZ conspicuity and segmentation quality between arterial and venous phases, with neither consistently providing superior imaging i.e., both phases offer complementary information. Timing also influenced appearances, where arterial phase was more often favoured < 30 min post-ablation, while venous phase dominated later due to diminishing arterial enhancement (Fig. 4). Ordinal regression quantified that arterial phase AZ conspicuity and segmentation quality score declined by 3 – 4% per minute that elapses after ablation. Practically speaking, our results support imaging as soon as possible after ablation. Thus, anticipatory preloading contrast injectors and connecting of cannulas may be prudent. Operators should also be cognisant that venous phase imaging is usually more useful for imaging of tumours treated earlier in the session. Regardless, beyond AZ depiction, biphasic imaging enables detailed characterisation of a greater range of potential complications including active bleeding, pseudoaneurysms and arteriovenous fistulas.

In a like-for-like comparison, we found that both AZ conspicuity and segmentation quality were significantly higher for the venous phase component of the bolus-tracked biphasic protocol versus fixed delay imaging; 4.0 vs. 2.5, *P* < 0.0001 for AZ conspicuity and 4.0 vs. 3.0, *P* = 0.002 for segmentation quality. Bolus tracking protocols improve consistency by reducing variation from cardiac output.

Our study provides insights into the biophysical basis of CT contrast in microwave AZs, which have three main components. Firstly, the central ‘charred zone’ of tissue carbonisation adjacent to the active antenna tip. Secondly, an inner ‘white zone’ of complete cell death [21]. Thirdly, an outer ‘red zone’ of incomplete cell death, hyperaemia, and inflammation [22]. Given the hyperaemic conditions of the red zone, it is highly plausible its imaging correlate is the hyperenhancing peripheral zone rim, supplied by the hepatic arteries, whereas the nonenhancing central AZ corresponds with the white zone [21–23].

Conspicuity is the linchpin for making all decisions regarding ablation adequacy, including quantitative assessments via segmentation. Accordingly, we prioritised conspicuity reliability in this study. While Mint Lesion™ is robustly validated [13, 24–26], further work that assesses AZ segmentation reliability is welcome. The single centre nature with exclusive focus on liver metastases in noncirrhotic livers is another limitation. Mixing retrospective and prospective cohorts introduces theoretical bias. However, there were no significant differences in baseline characteristics between cohorts, and there were no missing data. Furthermore, the primary outcome measure of image quality was analysed in the same fashion for both cohorts.

Future research should evaluate this optimal bolus tracking methodology on more diverse populations, examining how parenchyma (steatosis, fibrosis, cirrhosis) and tumour type affects image quality. Optimised imaging research could potentially be extended to quantitative ablation confirmation methodologies which consider the relationship between tumours and ablation zones. Moreover, emerging technologies including Transcatheter CT hepatic angiography [29–31], dual energy [32], perfusion [33, 34] and photon counting CT [35] hold the potential to further improve contrast resolution and should also be studied. Since not all CECT protocols are equal, minimum standards for scanning protocols could be defined, especially for multicentre trials [36]. Finally, to build upon our findings, our method could be developed to include fully automated segmentation techniques. By advancing automation and standardization in segmentation, the field can move closer to objective, reproducible assessments of ablation success, further mitigating subjectivity and allowing microwave liver ablation to reach its true potential.

## Conclusion

Bolus-tracked biphasic contrast-enhanced CT protocols dramatically improve both conspicuity and semi-automatic segmentation quality of microwave liver ablation zones, particularly if imaged as soon as possible after ablation. Adoption of such practices may improve and better standardise microwave liver ablation, by facilitating more accurate evaluation of ablative margins, and thus treatment success.
